# The Horse; Its External and Internal Organization

**Published:** 1895-08

**Authors:** 


### REVIEW.

THE HORSE; Its External and Internal Organization. Illustrated and descriptive. By A. SCHWARZ, Staff Veterinary Surgeon in the First Royal Bavarian Regiment of Light Horse. Revised and edited by George Fleming, F.R.C.V.S. Just issued from the publishing house of Thomas Whittaker, Fourth Avenue and Ninth Street, New York City.

This is a neat and concise book, specially destined to make clearer the usual instruction given under the head of farriery to the soldiers in the army. Also useful to convey to horsemen and horse-owners a clearer idea of the general conformation of the horse and the functions of the various organs and tissues. The five colored plates at the end will be found very interesting and instructive, and will be useful to the general practitioner, when desiring to convey quickly to his client a clear idea of any anatomical point.

